# BRCA mutation status and olaparib-related toxicity during maintenance therapy: a real-world retrospective cohort study

**DOI:** 10.3389/fonc.2026.1789787

**Published:** 2026-03-30

**Authors:** Yuewen Gao, Minxue Gai, Hongyang Zhang, Hongqi Li

**Affiliations:** 1 Department of Obstetrics and Gynecology, Union Hospital, Tongji Medical College, Huazhong University of Science and Technology, Wuhan, China; 2 Department of Gynecology, Shandong Provincial Hospital, Cheeloo College of Medicine, Shandong University, Jinan, Shandong, China

**Keywords:** adverse events, BRCA mutation, epithelial ovarian cancer, olaparib, PARP inhibitor, toxicity

## Abstract

**Objective:**

Olaparib is a standard maintenance therapy for epithelial ovarian cancer, particularly in patients with BRCA mutations. While BRCA status is an established predictive biomarker for efficacy, its potential impact on treatment-related toxicity remains uncertain. We evaluated the association between BRCA mutation status and the incidence and severity of olaparib-related adverse events in a real-world cohort.

**Method:**

This retrospective observational study included patients with epithelial ovarian cancer who received olaparib maintenance therapy at Shandong Provincial Hospital between August 2018 and October 2021. Patients were stratified by BRCA mutation status. Adverse events were graded according to the Common Terminology Criteria for Adverse Events (CTCAE) version 5.0 and analyzed at the patient level. Clinically relevant adverse events were defined as grade ≥2, as these typically require medical intervention, closer monitoring, or dose modification. Multivariable logistic regression was performed to adjust for potential confounders.

**Results:**

A total of 40 patients were included (24 BRCA-mutant and 16 BRCA wild-type). Clinically relevant adverse events (grade ≥2) occurred more frequently in the BRCA-mutant group than in the BRCA wild-type group (62.5% vs. 18.8%; OR 7.22, 95% CI 1.65–27.42; p = 0.0097). In multivariable analysis adjusting for age and prior chemotherapy cycles, BRCA mutation status remained significantly associated with grade ≥2 adverse events (OR 8.66, 95% CI 1.67–44.81; p = 0.010). Treatment discontinuation due to adverse events was uncommon in both groups.

**Conclusions:**

In this real-world retrospective cohort, BRCA mutation status was associated with increased risk of clinically significant olaparib-related toxicity. These findings suggest that BRCA mutation status may help identify patients at higher risk of adverse events and support closer toxicity monitoring and individualized dose management during maintenance therapy.

## Introduction

Ovarian cancer remains one of the most lethal gynecologic malignancies and is frequently diagnosed at an advanced stage due to the lack of specific early symptoms (1, 2). Although advances in cytoreductive surgery and platinum-based chemotherapy have improved outcomes, recurrence remains common, highlighting the need for effective maintenance strategies.

Poly(ADP-ribose) polymerase (PARP) inhibitors have become a cornerstone of maintenance therapy for epithelial ovarian cancer, particularly in tumors with homologous recombination repair deficiency, including BRCA1 and BRCA2 mutations (3, 4). Olaparib, the first PARP inhibitor approved for ovarian cancer, has demonstrated significant clinical benefit in both frontline and recurrent disease settings, as shown in pivotal trials such as SOLO-1 and PAOLA-1 (5, 6).

With the widespread adoption of olaparib, increasing attention has been directed toward treatment-related adverse events (AEs), which may affect treatment adherence, dose intensity, and quality of life. Common olaparib-related AEs include gastrointestinal symptoms and hematologic toxicities, and a proportion of patients require dose reduction or treatment discontinuation due to intolerance (7–9). In addition, recent reviews and long-term analyses have emphasized the importance of monitoring cumulative and late toxicities associated with prolonged PARP inhibitor exposure in real-world settings (10).

While BRCA mutation status is an established predictive biomarker for PARP inhibitor efficacy, its association with treatment-related toxicity remains incompletely understood. BRCA1 and BRCA2 are essential components of homologous recombination DNA repair in both tumor and normal tissues, raising the possibility that BRCA-mutant normal cells may exhibit increased susceptibility to PARP inhibitor–induced toxicity. Previous studies evaluating chemotherapy-related toxicity according to BRCA status have reported inconsistent findings, and differences in olaparib-related toxicity according to BRCA mutation status have often not been systematically analyzed. However, real-world data examining olaparib-related toxicity stratified by BRCA mutation status remain limited (11). Moreover, randomized clinical trials are conducted in highly selected patient populations under controlled conditions, which may not fully reflect toxicity patterns and management strategies encountered in routine clinical practice. Therefore, in this retrospective single-center study, we investigated the association between BRCA mutation status and the incidence and severity of olaparib-related adverse events in patients with epithelial ovarian cancer, using patient-level adverse event incidence and maximum CTCAE grade per patient.

## Methods

### Study design and patient population

This retrospective observational study was conducted at Shandong Provincial Hospital. Medical records of patients diagnosed with epithelial ovarian cancer who received olaparib maintenance therapy between August 2018 and October 2021 were reviewed. Eligibility criteria included: (1) histologically confirmed epithelial ovarian cancer; (2) receipt of olaparib as maintenance therapy following platinum-based chemotherapy; (3) known BRCA mutation status; and (4) availability of complete clinical and adverse event data. Patients with insufficient follow-up information or unclear BRCA mutation status were excluded.

A total of 40 patients met the inclusion criteria and were included in the final analysis. Patients were stratified into two groups according to BRCA mutation status: BRCA-mutant (n = 24) and BRCA wild-type (n = 16).

### Data collection

Clinical and pathological data were extracted from electronic medical records and supplemented by telephone follow-up when necessary. The following baseline variables were collected: age at initiation of olaparib therapy, Eastern Cooperative Oncology Group (ECOG) performance status, International Federation of Gynecology and Obstetrics (FIGO) stage, histological subtype, primary tumor location, receipt of neoadjuvant chemotherapy, number of platinum-based chemotherapy cycles, best response to chemotherapy, and post-chemotherapy cancer antigen 125 (CA-125) level, renal function(serum creatinine) and hepatic function(aspartate aminotransferase and alanine aminotransferase). BRCA mutation status was obtained from routine clinical testing records; mutations were classified as BRCA1 or BRCA2 and as germline or somatic according to the original clinical test reports.

Treatment-related data included duration of olaparib therapy, dose reduction, treatment discontinuation, and reasons for discontinuation (disease recurrence or progression, adverse events, or other reasons).

### Assessment of adverse events

Adverse events were identified through systematic review of electronic medical records, including outpatient clinic notes, hospitalization records, laboratory data, and physician documentation during olaparib therapy. When necessary, telephone follow-up was conducted to supplement or clarify adverse event information. Adverse events were attributed to olaparib based on documented clinical assessment by treating physicians and temporal association with treatment exposure. Events clearly attributable to disease progression or unrelated comorbid conditions were excluded when sufficient documentation was available. Adverse events were graded according to the Common Terminology Criteria for Adverse Events (CTCAE), version 5.0, which was applied uniformly during data extraction. Patients were routinely followed in outpatient clinics at approximately 4-week intervals during maintenance therapy, with laboratory monitoring performed according to institutional practice.

Adverse events were analyzed at the patient level. Each patient was counted once for each adverse event category if at least one event of that category occurred during olaparib treatment, regardless of the number or frequency of events. Adverse events were categorized as gastrointestinal, hematologic, or other adverse events based on clinical characteristics.

To assess overall toxicity burden, the maximum CTCAE grade per patient was recorded and used as a measure of adverse event severity. This patient-level approach captures the most clinically significant toxicity experienced during treatment and facilitates meaningful comparison of overall toxicity burden between groups. Clinically relevant adverse events were defined as adverse events of grade ≥2 or grade ≥3.

### Statistical analysis

Statistical analyses were performed using GraphPad Prism (GraphPad Software, San Diego, CA, USA). Continuous variables were summarized as medians with interquartile ranges (IQRs), and categorical variables were summarized as frequencies and percentages. Comparisons between the BRCA-mutant and BRCA wild-type groups were conducted using non-parametric statistical methods due to the small sample size. Continuous and ordinal variables were compared using the Mann–Whitney U test. Categorical variables were compared using Fisher’s exact test. To assess whether BRCA mutation status was independently associated with clinically relevant toxicity (grade ≥2 adverse events), multivariable logistic regression was performed including BRCA status, age, and number of prior platinum-based chemotherapy cycles as covariates. Given the limited number of events, additional covariates were not included to minimize model overfitting. Given the limited number of events (18 grade ≥2 events overall; EPV ≈ 6 for 3 predictors), additional covariates were not included to minimize model overfitting. All statistical tests were two-tailed, and a p value < 0.05 was considered statistically significant. There were no missing data for variables included in the primary analyses.

## Results

### Patient characteristics and olaparib treatment exposure

A total of 40 patients with histologically confirmed epithelial ovarian cancer who received olaparib maintenance therapy were included in the final analysis. According to BRCA mutation status, 24 patients were classified as BRCA-mutant and 16 as BRCA wild-type. Baseline demographic and clinicopathological characteristics were comparable between the two groups, including age, ECOG performance status, FIGO stage, histological subtype, primary tumor location, receipt of neoadjuvant chemotherapy, number of prior platinum-based chemotherapy cycles, response to chemotherapy, CA-125 level after chemotherapy, renal function and hepatic function (**Table 1**). Among the 24 BRCA-mutant patients, 18 (75.0%) had BRCA1 mutations and 6 (25.0%) had BRCA2 mutations; 20 (83.3%) were germline mutations and 4 (16.7%) were somatic mutations (**Supplementary Table 1**). Because of the small size of BRCA2 and somatic subgroups, BRCA-mutant patients were analyzed as a single group.

**Table 1:** Baseline demographic and clinicopathological characteristics of patients treated with olaparib.

Characteristic	BRCA-mutant (n = 24)	BRCA wild-type (n = 16)	P value
Age, years, median (range)	56.9 (42–74)	56.4 (34–70)	0.95
ECOG performance status, n (%)			0.999
0	16 (66.7)	10 (62.5)	
1	8 (33.3)	6 (37.5)	
FIGO stage, n (%)			0.44
I	1 (4.2)	2 (12.5)	
II	2 (8.3)	0 (0)	
III	13 (54.2)	11 (68.8)	
IV	8 (33.3)	3 (18.7)	
Primary tumor location, n (%)			0.64
Ovary	21 (87.5)	15 (93.8)	
Fallopian tube	3 (12.5)	1 (6.2)	
Histological type, n (%)			0.06
High-grade serous carcinoma	24 (100)	13 (81.3)	
Other	0 (0)	3 (18.7)	
Receipt of neoadjuvant chemotherapy, n (%)			0.26
Yes	8 (33.3)	2 (12.5)	
No	16 (66.7)	14 (87.5)	
Number of platinum-based chemotherapy cycles, n (%)			0.66
< 6	2 (8.3)	1 (6.3)	
6–8	18 (75.0)	10 (62.5)	
> 8	4 (16.7)	5 (31.2)	
Best response to platinum chemotherapy, n (%)			0.68
Complete response	19 (79.2)	14 (87.5)	
Partial response	5 (20.8)	2 (12.5)	
CA-125 level after chemotherapy, n (%)			0.68
≤ Upper limit of normal	19 (79.2)	14 (87.5)	
> Upper limit of normal	5 (20.8)	2 (12.5)	
Renal function			0.64
Normal	21	15	
Abnormal	3	1	
Hepatic function			0.11
Normal	17	15	
Abnormal	7	1	

Data are presented as number (%) unless otherwise indicated.

ECOG, Eastern Cooperative Oncology Group; FIGO, International Federation of Gynecology and Obstetrics.

P values were calculated using the chi-square test or Fisher’s exact test for categorical variables and the Mann–Whitney U test for continuous variables, as appropriate.

The median duration of olaparib treatment was 698 days (interquartile range [IQR], 615–759) in the BRCA-mutant group and 582 days (IQR, 323–833) in the BRCA wild-type group, with no statistically significant difference observed (p = 0.321). Dose reduction occurred in 3 patients (12.5%) in the BRCA-mutant group and in 1 patient (6.3%) in the BRCA wild-type group (p = 0.638).

At the time of last follow-up, 14 patients (35.0%) had discontinued olaparib therapy. Treatment discontinuation occurred in 6 patients (25.0%) in the BRCA-mutant group and in 8 patients (50.0%) in the BRCA wild-type group (p = 0.176). Disease recurrence or progression was the most common reason for discontinuation and occurred more frequently in the BRCA wild-type group than in the BRCA-mutant group (43.8% vs. 16.7%, p = 0.080). Discontinuation due to adverse events was uncommon and similar between the two groups (8.3% vs. 6.3%, p > 0.999) (**Table 2**).

**Table 2:** Olaparib treatment exposure and discontinuation.

Variable	BRCA-mutant (n=24)	BRCA wild-type (n=16)	P value
Treatment duration, days, median (IQR)	698 (615–759)	582 (323–833)	0.321
Dose reduction, n (%)	3 (12.5)	1 (6.3)	0.638
Treatment discontinuation, n (%)	6 (25.0)	8 (50.0)	0.176
Discontinuation due to recurrence/progression, n (%)	4 (16.7)	7 (43.8)	0.080
Discontinuation due to adverse events, n (%)	2 (8.3)	1 (6.3)	>0.999

### Incidence of olaparib-related adverse events

Adverse events were assessed and analyzed at the patient level. Overall, 22 of 24 patients (91.7%) in the BRCA-mutant group and 11 of 16 patients (68.8%) in the BRCA wild-type group experienced at least one olaparib-related adverse event of any grade during treatment, although this difference did not reach statistical significance (OR 5.00, 95% CI 0.78–27.08; p = 0.094).

**Figure 1:**
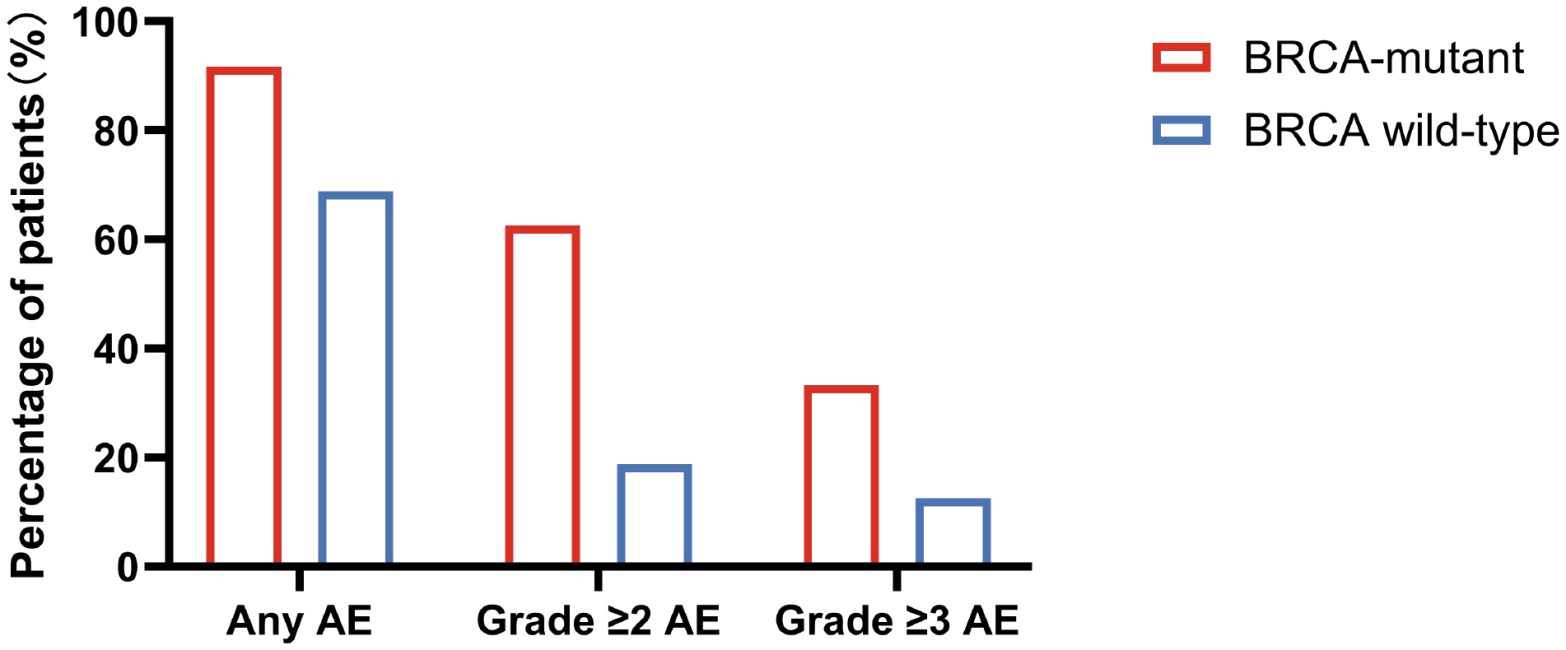
Incidence of olaparib-related adverse events stratified by BRCA mutation status.

The incidence of clinically relevant adverse events differed significantly between the two groups. Grade ≥2 adverse events occurred in 15 patients (62.5%) in the BRCA-mutant group compared with 3 patients (18.8%) in the BRCA wild-type group (ARD, 43.8 percentage points; OR 7.22, 95% CI 1.65–27.42; p = 0.0097). Grade ≥3 adverse events were observed in 8 patients (33.3%) in the BRCA-mutant group and in 2 patients (12.5%) in the BRCA wild-type group, but this difference was not statistically significant (OR 3.50, 95% CI 0.74–18.02; p = 0.263)(**Table 3**, **Figure 1**). In multivariable logistic regression analysis adjusting for age and prior platinum-based chemotherapy cycles, BRCA mutation status remained significantly associated with grade ≥2 adverse events (OR 8.66, 95% CI 1.67–44.81; p = 0.010). In contrast, age and chemotherapy cycles were not independently associated with clinically relevant toxicity (**Table 4**).

**Table 3:** Incidence of olaparib-related adverse events.

Adverse event category	BRCA-mutant (n=24)	BRCA wild-type (n=16)	P value
Any adverse event (≥ Grade 1)	22 (91.7%)	11 (68.8%)	0.094
Any adverse event (≥ Grade 2)	15 (62.5%)	3 (18.8%)	0.0097
Any adverse event (≥ Grade 3)	8 (33.3%)	2 (12.5%)	0.263
Gastrointestinal adverse events	17 (70.8%)	8 (50.0%)	0.205
Hematologic adverse events	12 (50.0%)	4 (25.0%)	0.188
Other adverse events	6 (25.0%)	5 (31.2%)	0.728

**Table 4:** Multivariable logistic regression for grade ≥2 adverse events.

Adverse event category	OR	95%CI	P value
BRCA-mutant vs wild-type	8.66	1.67–44.81	0.010
Age	-	-	0.113
Platinum cycles	-	-	0.843

Gastrointestinal adverse events were the most frequently reported toxicities in both groups, occurring in 70.8% of BRCA-mutant patients and 50.0% of BRCA wild-type patients (p = 0.205). Hematologic adverse events were observed in 50.0% of patients in the BRCA-mutant group and in 25.0% of patients in the BRCA wild-type group (p = 0.188). Other adverse events, including fatigue, rash, and peripheral neuropathy, occurred at similar frequencies between the two groups (25.0% vs. 31.2%, p = 0.728) (**Table 3**, **Figure 1**). Detailed frequencies of specific adverse event types stratified by BRCA status are provided in **Supplementary Table 2**.

Adverse event incidence was calculated at the patient level. Clinically relevant adverse events were defined as adverse events of grade ≥2 or grade ≥3 according to the Common Terminology Criteria for Adverse Events (CTCAE), version 5.0. BRCA-mutant patients demonstrated a higher incidence of grade ≥2 adverse events compared with BRCA wild-type patients.

### Severity of olaparib-related adverse events

To evaluate overall toxicity burden, the maximum CTCAE grade per patient was used as a measure of adverse event severity. In the BRCA-mutant group, 2 patients experienced no adverse events, 7 experienced a maximum grade 1 event, 7 experienced a maximum grade 2 event, and 8 experienced a maximum grade 3 event. In contrast, among BRCA wild-type patients, 5 experienced no adverse events, 8 experienced a maximum grade 1 event, 1 experienced a maximum grade 2 event, and 2 experienced a maximum grade 3 event.

**Figure 2:**
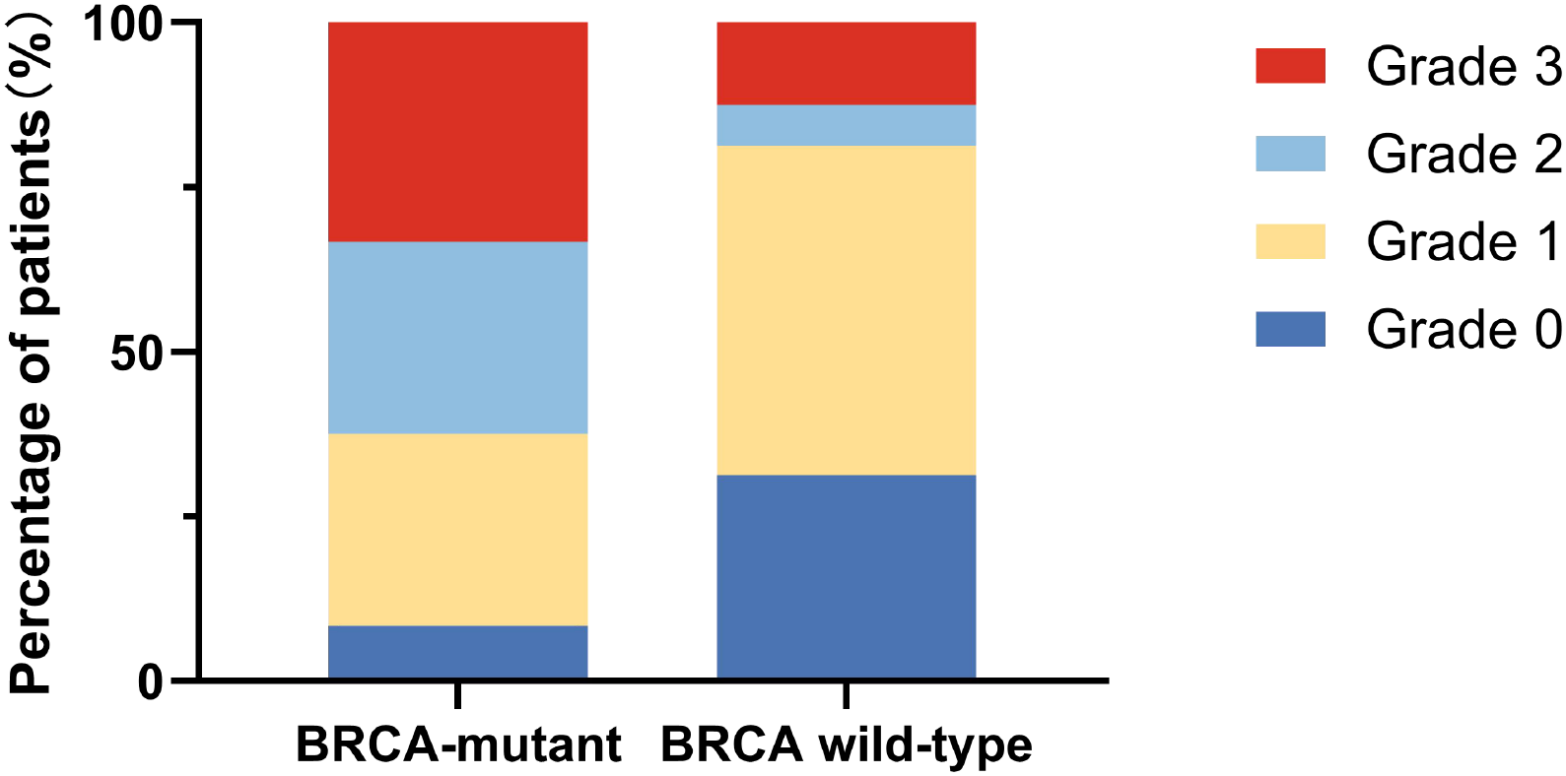
Distribution of the maximum olaparib-related adverse event grade per patient stratified by BRCA mutation status.

The distribution of maximum adverse event grades differed significantly between the two groups, with BRCA-mutant patients experiencing greater overall toxicity severity compared with BRCA wild-type patients (Mann–Whitney U test, p = 0.0085) (**Table 5**,**Figure 2**).

**Table 5:** Maximum adverse event grade per patient.

Maximum CTCAE grade	BRCA-mutant (n=24)	BRCA wild-type (n=16)
Grade 0	2 (8.3%)	5 (31.3%)
Grade 1	7 (29.2%)	8 (50.0%)
Grade 2	7 (29.2%)	1 (6.3%)
Grade 3	8 (33.3%)	2 (12.5%)

Adverse events were graded according to the Common Terminology Criteria for Adverse Events (CTCAE), version 5.0. Each patient was classified according to the highest-grade adverse event experienced during olaparib treatment. BRCA-mutant patients exhibited a higher proportion of grade 2–3 adverse events compared with BRCA wild-type patients.

### Summary of safety findings

Taken together, these findings indicate that while olaparib maintenance therapy was generally tolerable in both groups, BRCA mutation status was associated with increased adverse event severity and a higher incidence of clinically significant (grade ≥2) toxicities. These differences were observed despite comparable treatment duration and similar rates of dose modification between BRCA-mutant and BRCA wild-type patients.

## Discussion

In this retrospective real-world cohort of epithelial ovarian cancer patients receiving olaparib maintenance therapy, BRCA mutation status was associated with increased toxicity severity. Specifically, BRCA-mutant patients experienced a significantly higher incidence of clinically relevant adverse events (grade ≥2) and greater overall toxicity burden, as reflected by the maximum CTCAE grade per patient. This association remained significant after adjustment for age and prior chemotherapy exposure, supporting the robustness of the observed relationship. Importantly, the increased incidence of adverse events in BRCA-mutant patients likely reflects greater biological susceptibility to olaparib-related toxicity rather than true treatment intolerance. Despite the higher frequency of clinically relevant adverse events, treatment discontinuation due to toxicity was uncommon and comparable between groups, indicating that most toxicities were manageable with appropriate supportive care and dose modifications. These findings suggest that BRCA mutation status may influence not only the efficacy but also the tolerability of olaparib in routine clinical practice.

The safety profile of olaparib has been well characterized in randomized clinical trials, in which gastrointestinal and hematologic toxicities are consistently reported as the most frequent adverse events (5–9). In SOLO-1, the most common grade 3–4 adverse events in the olaparib arm included anemia (~21–22%) and neutropenia (~8%), and dose interruption, dose reduction, and treatment discontinuation due to adverse events occurred in approximately 52%, 28%, and 12% of patients, respectively (5). Similarly, in SOLO-2, grade ≥3 toxicities included anemia (~19%), fatigue/asthenia (~4%), and neutropenia (~5%), with treatment interruption, dose reduction, and discontinuation rates of approximately 50%, 28%, and 17%, respectively (12). In our cohort, grade ≥3 adverse events occurred in 33.3% of BRCA-mutant patients and 12.5% of BRCA wild-type patients, with low discontinuation rates in both groups. Real-world cohorts have reported variable toxicity and discontinuation rates; for example, one recent study reported discontinuation due to toxicity of ~1.4% in first-line maintenance and ~6.0% in later-line settings (13). Notably, pivotal trials such as SOLO-1 and SOLO-2 were conducted in highly selected patient populations under strict protocol-driven monitoring and management strategies. In contrast, real-world practice includes greater patient heterogeneity, comorbidities, and less standardized supportive care, which may contribute to differences in toxicity patterns and reporting. These contextual differences underscore the importance of real-world data in understanding the clinical implications of BRCA mutation status on olaparib tolerability.

A biologically plausible explanation for the increased toxicity severity observed in BRCA-mutant patients lies in the role of BRCA1 and BRCA2 in homologous recombination DNA repair. Germline BRCA mutations may impair DNA repair capacity not only in tumor cells but also in normal tissues such as bone marrow and gastrointestinal epithelium (14, 15). In patients initiating olaparib after platinum-based chemotherapy, residual subclinical tissue injury may further predispose BRCA-mutant individuals to treatment-related toxicity (16–20). Although prior studies evaluating chemotherapy-related toxicity by BRCA status have yielded inconsistent results (21–23), our findings suggest that this susceptibility may become more apparent during PARP inhibitor maintenance therapy.

Importantly, we analyzed adverse events at the patient level rather than event level, thereby minimizing bias related to repeated events in individual patients. We focused on clinically relevant toxicity by emphasizing grade ≥2 adverse events, as grade 1 toxicities are typically mild and rarely require treatment modification. This approach is consistent with clinical practice and prior safety analyses of PARP inhibitors (8).

Several limitations should be acknowledged. This was a single-center retrospective study with a modest sample size, which limits generalizability. Although multivariable analysis was performed, the limited number of events restricted the number of covariates that could be included without risking model overfitting. The wide confidence interval observed in the adjusted analysis reflects limited precision of the effect estimate, and residual confounding cannot be excluded. Based on the observed proportions, the study had approximately 82% power to detect the difference in grade ≥2 adverse events but substantially lower power (approximately 35%) for grade ≥3 events; therefore, non-significant findings for grade ≥3 toxicity should be interpreted cautiously, as Type II error remains possible. Although BRCA mutations were characterized as BRCA1 versus BRCA2 and as germline versus somatic, subgroup analyses were not performed due to limited sample size. Homologous recombination deficiency beyond BRCA status was not assessed. Adverse events were collected through systematic medical record review supplemented by telephone follow-up; however, retrospective ascertainment may be subject to incomplete documentation and potential surveillance bias. Detailed exposure–toxicity modeling and formal time-to-event analyses were not feasible in this cohort. In addition, the relatively short follow-up period precluded evaluation of rare late toxicities such as myelodysplastic syndrome and acute myeloid leukemia, which have been increasingly reported in long-term analyses (10, 24).

Despite these limitations, this study provides clinically relevant real-world evidence that BRCA mutation status is associated with increased severity of olaparib-related adverse events. While toxicities were generally manageable, BRCA-mutant patients may benefit from closer monitoring and proactive supportive care during maintenance therapy. These findings support a more individualized approach to toxicity surveillance and dose management in routine clinical practice. Future prospective, multicenter studies with longer follow-up and more detailed genomic characterization are needed to confirm these results and to further refine risk-adapted strategies for optimizing the safety of PARP inhibitor therapy.

## Data Availability

The raw data supporting the conclusions of this article will be made available by the authors, without undue reservation.

## References

[1] Konstantinopoulos PA Matulonis UA . Clinical and translational advances in ovarian cancer therapy. Nat Cancer. (2023) 4:1239–57. doi: 10.1038/s43018-023-00617-9 37653142

[2] Kuroki L Guntupalli SR . Treatment of epithelial ovarian cancer. BMJ. (2020) 371:m3773. doi: 10.1136/bmj.m3773 33168565

[3] Pujade-Lauraine E Ledermann JA Selle F Gebski V Penson RT Oza AM . Olaparib tablets as maintenance therapy in patients with platinum-sensitive, relapsed ovarian cancer and a BRCA1/2 mutation (SOLO2/ENGOT-Ov21): a double-blind, randomised, placebo-controlled, phase 3 trial. Lancet Oncol. (2017) 18:1274–84. doi: 10.1016/S1470-2045(17)30469-2, PMID: 28754483

[4] Ledermann J Harter P Gourley C Friedlander M Vergote I Rustin G . Olaparib maintenance therapy in platinum-sensitive relapsed ovarian cancer. N Engl J Med. (2012) 366:1382–92. doi: 10.1056/NEJMoa1105535, PMID: 22452356

[5] Moore K Colombo N Scambia G Kim BG Oaknin A Friedlander M . Maintenance olaparib in patients with newly diagnosed advanced ovarian cancer. N Engl J Med. (2018) 379:2495–505. doi: 10.1056/NEJMoa1810858, PMID: 30345884

[6] Ray-Coquard I Pautier P Pignata S Pérol D González-Martín A Berger R . Olaparib plus bevacizumab as first-line maintenance in ovarian cancer. N Engl J Med. (2019) 381:2416–28. doi: 10.1056/NEJMoa1911361, PMID: 31851799

[7] LaFargue CJ Dal Molin GZ Sood AK Coleman RL . Exploring and comparing adverse events between PARP inhibitors. Lancet Oncol. (2019) 20:e15–28. doi: 10.1016/S1470-2045(18)30786-1 30614472 PMC7292736

[8] Tian X Chen L Gai D He S Jiang X Zhang N . Adverse event profiles of PARP inhibitors: analysis of spontaneous reports submitted to FAERS. Front Pharmacol. (2022) 13:851246. doi: 10.3389/fphar.2022.851246, PMID: 35401230 PMC8990839

[9] Monk BJ González-Martín A Buckley L Matulonis UA Rimel BJ Wu X . Safety and management of niraparib monotherapy in ovarian cancer clinical trials. Int J Gynecol Cancer. (2023) 33:971–981. doi: 10.1136/ijgc-2022-004079, PMID: 36792166 PMC10313963

[10] O’Malley DM Krivak TC Kabil N Munley J Moore KN . PARP inhibitors in ovarian cancer: a review. Target Oncol. (2023) 18:471–503. doi: 10.1007/s11523-023-00970-w 37268756 PMC10344972

[11] Tuninetti V Marín-Jiménez JA Valabrega G Ghisoni E . Long-term outcomes of PARP inhibitors in ovarian cancer: survival, adverse events, and post-progression insights. ESMO Open. (2024) 9:103984. doi: 10.1016/j.esmoop.2024.103984 39541620 PMC11613435

[12] Poveda A Floquet A Ledermann JA Asher R Penson RT Oza AM . Olaparib tablets as maintenance therapy in patients with platinum-sensitive relapsed ovarian cancer and a BRCA1/2 mutation (SOLO2/ENGOT-Ov21): a final analysis of a double-blind, randomised, placebo-controlled, phase 3 trial. Lancet Oncol. (2021) 22:620–631. doi: 10.1016/S1470-2045(21)00073-5, PMID: 33743851

[13] Kim J Park SY Kim JH Lee SW Park JY Kim JH . Real-world efficacy and toxicity of olaparib maintenance therapy in Korean ovarian cancer patients with an exploratory analysis of BRCA mutations. Gynecol Oncol. (2025) 194:25–32. doi: 10.1016/j.ygyno.2025.01.013, PMID: 39923681

[14] Curtin NJ Szabo C . Poly(ADP-ribose) polymerase inhibition: past, present and future. Nat Rev Drug Discov. (2020) 19:711–36. doi: 10.1038/s41573-020-0076-6 32884152

[15] Lightfoot M Montemorano L Bixel K . PARP inhibitors in gynecologic cancers: what is the next big development? Curr Oncol Rep. (2020) 22:29. doi: 10.1007/s11912-020-0873-4 32067102

[16] Onstad M Coleman RL Westin SN . Movement of poly-ADP ribose (PARP) inhibition into frontline treatment of ovarian cancer. Drugs. (2020) 80:1525–35. doi: 10.1007/s40265-020-01382-0 32852746 PMC7541632

[17] Tomao F Musacchio L Di Mauro F Boccia SM Di Donato V Giancotti A . Is BRCA mutational status a predictor of platinum-based chemotherapy-related hematologic toxicity in high-grade serous ovarian cancer patients?. Gynecol Oncol. (2019) 154:138–143. doi: 10.1016/j.ygyno.2019.04.009, PMID: 31079832

[18] Weitzner O Yagur Y Kadan Y Beiner ME Fishman A Ben Ezry E . Chemotherapy toxicity in BRCA mutation carriers undergoing first-line platinum-based chemotherapy. Oncologist. (2019) 24:e1471–e1475. doi: 10.1634/theoncologist.2019-0272, PMID: 31346131 PMC6975939

[19] Lee IH Lee SJ Kim J Lee YH Chong GO Kim JM . Exploring the effect of BRCA1/2 status on chemotherapy-induced hematologic toxicity in patients with ovarian cancer. Cancer Chemother Pharmacol. (2024) 94:103–8. doi: 10.1007/s00280-024-04670-8, PMID: 38652271

[20] Friedlaender A Vuilleumier A Viassolo V Ayme A De Talhouet S Combes JD . BRCA1/BRCA2 germline mutations and chemotherapy-related hematological toxicity in breast cancer patients. Breast Cancer Res Treat. (2019) 174:775–783. doi: 10.1007/s10549-018-05127-2, PMID: 30635808

[21] Kotsopoulos J Willows K Trat S Kim RH Volenik A Sun P . BRCA mutation status is not associated with increased hematologic toxicity among patients undergoing platinum-based chemotherapy for ovarian cancer. Int J Gynecol Cancer. (2018) 28:69–76. doi: 10.1097/IGC.0000000000001144, PMID: 29194191

[22] Shanley S McReynolds K Ardern-Jones A Ahern R Fernando I Yarnold J . Acute chemotherapy-related toxicity is not increased in BRCA1 and BRCA2 mutation carriers treated for breast cancer. Clin Cancer Res. (2006) 12:7033–7038. doi: 10.1158/1078-0432.CCR-06-1246, PMID: 17145825

[23] Shanley S McReynolds K Ardern-Jones A Ahern R Fernando I Yarnold J . Late toxicity is not increased in BRCA1/BRCA2 mutation carriers undergoing breast radiotherapy. Clin Cancer Res. (2006) 12:7025–32. doi: 10.1158/1078-0432.CCR-06-1244, PMID: 17145824

[24] Corazón Villanueva J González Pérez C Arenaza Peña A Marquina G Casado Herraez A Sánchez-Del Hoyo R . Real-world incidence and risk of myelodysplastic syndrome and acute myeloid leukemia secondary to PARP inhibitors in ovarian cancer. Clin Transl Oncol. (2025). doi: 10.1007/s12094-025-04144-0, PMID: 41351780

