# Supplementary material for: BRCA mutation status and olaparib-related toxicity during maintenance therapy: a real-world retrospective cohort study
Source: Front Oncol. 2026 Mar 30;16:1789787. doi: 10.3389/fonc.2026.1789787 (PMC13070956; doi:10.3389/fonc.2026.1789787)
Supplement: Supplementary file 1 [file Table1.docx]

**Table S1. BRCA mutation characteristics in the BRCA-mutant group (n=24)**

| **Characteristic** | **n(%)** |
| --- | --- |
| BRCA gene | |
| BRCA1 | 18 (75.0) |
| BRCA2 | 6 (25.0) |
| Mutation origin | |
| Germline | 20 (83.3) |
| Somatic | 4 (16.7) |
