# Supplementary material for: BRCA mutation status and olaparib-related toxicity during maintenance therapy: a real-world retrospective cohort study
Source: Front Oncol. 2026 Mar 30;16:1789787. doi: 10.3389/fonc.2026.1789787 (PMC13070956; doi:10.3389/fonc.2026.1789787)
Supplement: Supplementary file 2 [file Table2.docx]

**Table S2. Specific olaparib-related adverse events by BRCA status (patient-level incidence)**

| **Specific AE** | **BRCA-mutant (n=24)** | **BRCA wild-type (n=16)** |
| --- | --- | --- |
| Nausea/vomiting | 17/24 (70.8%) | 8/16 (50.0%) |
| Anemia | 12/24 (50.0%) | 4/16 (25.0%) |
| Fatigue | 5/24 (20.8%) | 2/16 (12.5%) |
| Neutropenia | 2/24 (8.3%) | 0/16 (0.0%) |
| Rash | 1/24 (4.2%) | 1/16 (6.2%) |
| Peripheral neuropathy | 0/24 (0.0%) | 2/16 (12.5%) |
